# Fat‐specific protein 27α inhibits autophagy‐dependent lipid droplet breakdown in white adipocytes

**DOI:** 10.1111/jdi.13050

**Published:** 2019-04-26

**Authors:** Shinsuke Nakajima, Yuki Nishimoto, Sanshiro Tateya, Yasuyuki Iwahashi, Yuko Okamatsu‐Ogura, Masayuki Saito, Wataru Ogawa, Yoshikazu Tamori

**Affiliations:** ^1^ Department of Internal Medicine Division of Diabetes and Endocrinology Kobe University Graduate School of Medicine Kobe Japan; ^2^ Department of Internal Medicine Division of Diabetes Kakogawa Central City Hospital Kakogawa Japan; ^3^ Department of Biomedical Sciences Graduate School of Veterinary Medicine Hokkaido University Sapporo Japan; ^4^ Department of Social/Community Medicine and Health Science Division of Creative Health Promotion Kobe University Graduate School of Medicine Kobe Japan

**Keywords:** Autophagy, Fat‐specific protein 27, White adipocytes

## Abstract

**Aims/Introduction:**

Fat‐specific protein 27 (FSP27) α is the major isoform of FSP27 in white adipose tissue (WAT), and is essential for large unilocular lipid droplet (LD) formation in white adipocytes. In contrast, FSP27β is abundantly expressed in brown adipose tissue (BAT), and plays an important role in small multilocular LD formation. In FSP27 KO mice in which FSP27α and β are both depleted, WAT is characterized by multilocular LD formation, and by increased mitochondrial abundance and energy expenditure, whereas BAT conversely manifests large oligolocular LDs and reduced energy expenditure.

**Materials and Methods:**

We investigated the effects of autophagy in WAT and BAT of wild type (WT) and FSP27 knockout (KO) mice. In addition, we examined the effects of FSP27α and FSP27β to the induction of autophagy in COS cells.

**Results:**

Food deprivation induced autophagy in BAT of WT mice, as well as in WAT of FSP27 KO mice, suggesting that enhanced autophagy is characteristic of adipocytes with small multilocular LDs. Pharmacological inhibition of autophagy attenuated the fasting‐induced loss of LD area in adipocytes with small multilocular LDs (BAT of WT mice and WAT of FSP27 KO mice), without affecting that in adipocytes with large unilocular or oligolocular LDs (WAT of WT mice or in BAT of FSP27 KO mice). Overexpression of FSP27α inhibited autophagy induction by serum deprivation in COS cells, whereas that of FSP27β had no such effect.

**Conclusions:**

The present results thus showed that FSP27α inhibits autophagy and might thereby contribute to the energy‐storage function of WAT.

## Introduction

Mammals possess two main types of adipose tissue with distinct functions. White adipose tissue (WAT) stores energy in the form of triglyceride (TG) for expenditure during periods of food deprivation, whereas brown adipose tissue (BAT) consumes stored energy for heat production in a cold environment^1^. These two types of adipose tissue also differ in the morphology of intracellular lipid droplets (LDs), which are large and unilocular in WAT, and small and multilocular in BAT^2^, with this difference likely reflecting the metabolic characteristics of the adipocytes.

The α isoform of fat‐specific protein 27 (FSP27α), which belongs to the cell death‐inducing deoxyribonucleic acid (DNA) fragmentation factor A‐like effector (Cide) family of proteins, has been shown to be indispensable for large unilocular LD formation in WAT^3^, ^4^, ^5^, ^6^. We also recently found that FSP27β, a novel isoform of FSP27 that is abundant in BAT, plays a key role in small multilocular LD formation in this tissue by inhibiting the homodimerization of CideA^7^. FSP27α and FSP27β are the splice variants driven by distinct promoters from the same gene^8^. Thus, FSP27α and FSP27β are both deficient in the FSP27 knockout (KO) mice we produced^3^. In FSP27 KO mice, WAT is characterized by multilocular LD formation, and by increased mitochondrial abundance and energy expenditure, whereas BAT conversely manifests large oligolocular LDs, and reduced mitochondrial abundance and energy expenditure^3^. Unilocular LD formation might contribute to efficient lipid storage in WAT, because lipolysis from the LD surface is restricted by the minimal surface area, with the free fatty acids (FFAs) and glycerol generated by TG hydrolysis then entering the circulation for transport to other tissues. In contrast, small multilocular LD formation might promote efficient intracellular lipolysis from the LD surface and subsequent transport of FFAs to adjacent mitochondria for β‐oxidation in BAT. Intracellular LD morphology and lipolysis are thus closely related to the functions and characteristics of the different types of adipose tissue^2^.

Hydrolysis of TG in LDs is generally mediated by cytosolic lipases^9^. However, LDs also serve as a substrate for macroautophagy. Autophagy is a lysosomal degradative pathway for the removal and breakdown of cellular components, such as organelles and proteins, that are especially important during periods of food deprivation. Lipolysis and autophagy share certain similarities, with both processes being important for adaptation to nutrient deprivation. In addition, autophagy has been shown to play an important role in lipid metabolism and storage^10^. For example, inhibition of autophagy was found to increase lipid accumulation in the liver of mice^11^, whereas activation of autophagy reduced hepatocellular lipid accumulation and lipotoxicity^12^. Autophagy contributes to the regulation of adipocyte differentiation^13^, ^14^ and the beige‐to‐white fat transition^15^, but it has remained unclear whether it also plays a role in lipid metabolism in mature adipocytes. We have now investigated the possible contribution of autophagy to lipid degradation in WAT, which supplies FFAs to other tissues during food deprivation, as well as to that in BAT, which metabolizes FFAs during exposure to cold environments.

## Methods

### Quantitative reverse transcription polymerase chain reaction analysis

Total ribonucleic acid (RNA) extracted from adipose tissues was subjected to reverse transcription (RT) with the use of an RNeasy kit (Qiagen, Hilden, Germany), and the resulting complementary DNA was subjected to real‐time polymerase chain reaction (PCR) analysis with specific primers (Table 1) and Power SYBR Green PCR Master Mix (Applied Biosystems, Waltham, MA, USA) in a Sequence Detector (model 7500; PE Applied Biosystems). The abundance of target messenger RNAs (mRNAs) was normalized by that of 36B4 mRNA as the invariant control.

**Table 1:** Primer sequences for quantitative reverse transcription polymerase chain reaction analysis

mRNA	Sense (5′→3′)	Antisense (5′→3′)
Atg7	TCCGTTGAAGTCCTCTGCTT	CCACTGAGGTTCACCATCCT
p62	TGTGGAACATGGAGGGAAGAG	TGTGCCTGTGCTGGAACTTTC
Ulk1	AGATTGCTGACTTTGGATTC	AGCCATGTACATAGGAGAAC
Gabarapl1	TCGTGGAGAAGGCTCCTAAA	ATACAGCTGGCCCATGGTAG
AMPKα1	GCTCACCCAACTATGCTGCAC	TATCTACCTCTGGGCCTGCATACAA
Sirt1	GACGGTATCTATGCTCGCCT	ATTCCTGCAACCTGCTCCAAG
FOXO1	ACATTTCGTCCTCGAACCAGCTCA	ATTTCAGACAGACTGGGCAGCGTA
ATGL	GGAGACCAAGTGGAACATCTCA	AATAATGTTGGCACCTGCTTCA
HSL	TGTGGCACAGACCTCTAAAT	GGCATATCCGCTCTC
LAL	GACCACTCCCGATGCAACTC	GACCACTCCTTGTGAGCCAG

AMPKα1, adenosine monophosphate‐activated protein kinase‐α; Atg7, autophagy‐related protein 7; ATGL, adipose triacylglycerol lipase; FOXO1, forkhead box protein O1; Gabarapl1, GABA type A receptor‐associated protein like 1; HSL, hormone‐sensitive lipase; LAL, lysosomal acid lipase; mRNA, messenger ribonucleic acid; Sirt1, silent mating type information regulation 2 homolog 1; Ulk1, Unc51‐like kinase 1.

### Cell culture

White adipocyte (HW) and brown adipocyte (HB2) cells were prepared from epididymal WAT (eWAT) and interscapular BAT, respectively, of homozygous p53 KO mice, as described previously^16^. The cells were maintained in Dulbecco's modified Eagle's medium (Sigma‐Aldrich, St. Louis, MO, USA) supplemented with 10% fetal bovine serum (Gibco, Waltham, MA, USA), streptomycin (50 μg/mL) and penicillin (50 U/mL). For induction of adipocyte differentiation, complete medium supplemented with 0.5 mmol/L 3‐isobutyl‐1‐methylxanthine and 1 μmol/L dexamethasone was added to the cells at confluence. After 2 days, the medium was changed to Dulbecco's modified Eagle's medium supplemented with 50 nmol/L triiodothyronine and insulin (10 μg/mL), and was refreshed every 2 days. The cells were subjected to experiments at 6–8 days after the onset of differentiation induction. COS cells were maintained in Dulbecco's modified Eagle's medium supplemented with 10% fetal bovine serum.

### Immunoblot analysis

Tissue homogenates were prepared with the use of a Teflon homogenizer in a lysis buffer containing 50 mmol/L Tris‐HCl (pH 8.0), 250 mmol/L NaCl, 1% Nonidet P40, 0.5% sodium deoxycholate, 0.1% sodium dodecyl sulfate, 1 mmol/L ethylenediaminetetraacetic acid, 50 mmol/L sodium fluoride, 10 mmol/L sodium pyrophosphate, 1 mmol/L sodium vanadate, 1 mmol/L phenylmethylsulfonyl fluoride and 1% protease inhibitors (Sigma‐Aldrich). Lysates of HW, HB2 or COS cells were prepared with a lysis buffer containing 25 mmol/L Tris‐HCl (pH 7.4), 150 mmol/L NaCl, 1% Nonidet P40, 1 mmol/L ethylenediaminetetraacetic acid, 50 mmol/L NaF, 10 mmol/L sodium pyrophosphate, 1 mmol/L sodium vanadate, 1 mmol/L phenylmethylsulfonyl fluoride and 1% protease inhibitors. Primary antibodies for immunoblot analysis included rabbit polyclonal antibodies to FSP27 generated as described previously^3^; rabbit antibodies to p62 and to Beclin1, as well as mouse antibodies to LC3 from Medical & Biological Laboratories (Aichi, Japan); rabbit antibodies to forkhead box protein O1 (FOXO1) and to total or phosphorylated adenosine monophosphate‐activated protein kinase‐α (AMPKα) from Cell Signaling Technology (Danvers, MA, USA); and mouse antibodies to α‐tubulin and to β‐actin from Sigma‐Aldrich. Immune complexes were detected with horseradish peroxidase‐conjugated secondary antibodies and enhanced chemiluminescence reagents (GE Healthcare, Chicago, IL, USA).

### Histological analysis

Epididymal WAT, subcutaneous WAT (sWAT) and interscapular BAT of mice were fixed with 10% formalin, embedded in paraffin, sectioned at a thickness of 6 μm and mounted on glass slides according to standard procedures. The sections were stained with hematoxylin–eosin and were examined with a BZ‐X710 microscope (Keyence, Osaka, Japan) for determination of LD area.

### Inhibition of autophagy in mice

FSP27 KO mice were generated as described previously^3^. The mice used in the present study are all male. Mice at 12 weeks of age were injected intraperitoneally with the lysosomal protease inhibitor, leupeptin (40 mg/kg), at 0, 4, 8 and 12 h after the onset of food deprivation, and were analyzed at 14 h. All mouse experiments were approved by the Animal Ethics Committee of Kobe University Graduate School of Medicine (Registration No: A170314).

### Overexpression of FSP27 and immunofluorescence analysis in COS cells

Full‐length complementary DNAs for mouse FSP27α or FSP27β were subcloned into the expression plasmid pIRES2‐DsRed2 (Clontech, Mountain View, CA, USA) 7, and the resulting vectors were introduced into COS cells with the use of the X‐tremeGENE 9 DNA Transfection Reagent (Roche Applied Science, Penzberg, Upper Bavaria, Germany). The cells were subsequently fixed with phosphate‐buffered saline (PBS) containing 4% paraformaldehyde at room temperature, washed with PBS and exposed to PBS containing 5% bovine serum albumin. For immunostaining of LC3, the cells were permeabilized with 0.2% Triton X‐100 for 5 min, exposed to PBS containing 5% bovine serum albumin, and then incubated consecutively with rabbit antibodies to LC3 (Medical & Biological Laboratories) and Dylight405‐conjugated goat antibodies to rabbit immunoglobulin G (ThermoScientific, Waltham, MA, USA). The cells were finally examined with a confocal laser‐scanning microscope (BZ‐X710; Keyence).

### Statistical analysis

Quantitative data are presented as the mean + standard error of the mean, and were compared between two groups with the use of the two‐tailed Student's *t*‐test. A *P*‐value of <0.05 was considered statistically significant.

## Results

### Autophagy is increased in WAT, but attenuated in BAT of FSP27 KO mice

**Figure 1:**
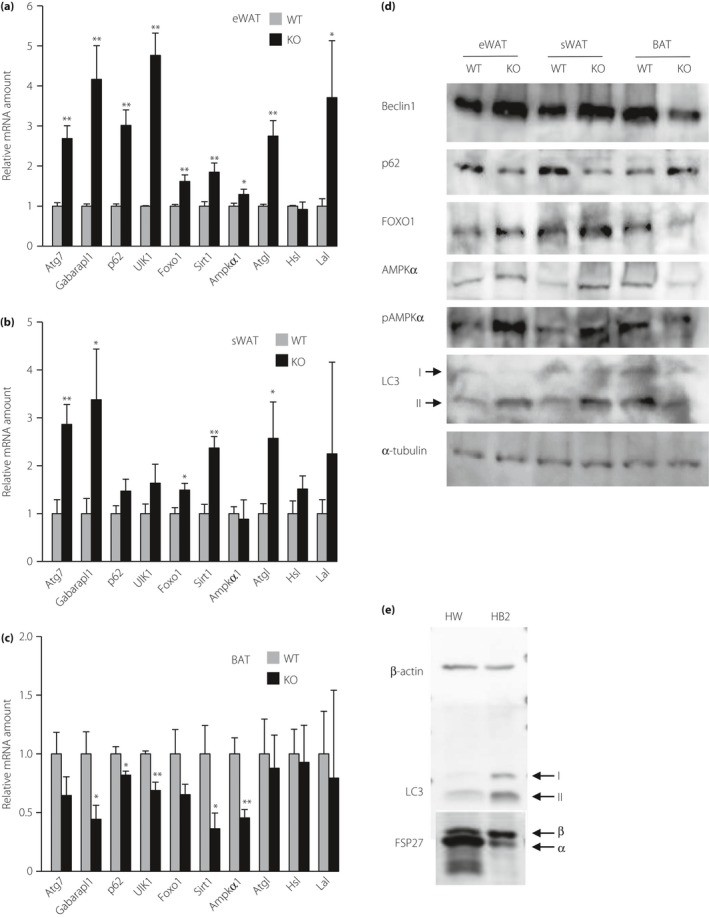
Enhancement of autophagy in white adipose tissue (WAT) of fat‐specific protein 27 (FSP27) knockout (KO) mice and brown adipose tissue (BAT) of wild‐type (WT) mice. (a–c) Quantitative reverse transcription polymerase chain reaction analysis of messenger ribonucleic acids (mRNAs) for autophagy‐related proteins in (a) epididymal WAT (eWAT), (b) subcutaneous WAT (sWAT) and (c) BAT of WT or FSP27 KO mice at 12 weeks of age that had been deprived of food for 14 h. Data were normalized by the amount of 36B4 mRNA, are expressed relative to the corresponding value for WT mice and are the mean + standard error of the mean (*n* = 6 mice per genotype). *P <* *0.05, **P <* *0.01 versus the corresponding value for WT mice. (d) Immunoblot analysis of autophagy‐related proteins and α‐tubulin (loading control) in eWAT, sWAT and BAT of 12‐week‐old WT or FSP27 KO mice that had been deprived of food for 14 h. (e) Immunoblot analysis of LC3, FSP27 and β‐actin (loading control) in white adipocyte (HW) and brown adipocyte (HB2) cells cultured in serum‐free medium for 40 h. AMPK, adenosine monophosphate‐activated protein kinase‐α; FOXO1, forkhead box protein O1.

We first examined the expression of autophagy‐related genes in eWAT, sWAT and BAT of food‐deprived FSP27 KO and WT mice by quantitative RT–PCR analysis. The amounts of mRNAs for autophagy‐related protein 7 (Atg7)^17^, the autophagy regulator Gabarapl1^18^, the autophagy mediator FOXO1^19^ and SIRT1, which mediates histone deacetylation at autophagy‐related genes^20^, were all significantly increased in eWAT and sWAT of FSP27 KO mice compared with WT mice (Figure 1a,b). The expression of genes for p62, an adaptor protein that functions coordinately with the autophagy mediator LC3^21^; for the autophagy initiator Unc51‐like kinase 1 (Ulk1)^22^; and for AMPKα1, which promotes autophagy by activating Ulk1 through direct phosphorylation^23^, was also increased in eWAT of FSP27 KO mice (Figure 1a). In BAT, however, the abundance of Gabarapl1, p62, Ulk1, Sirt1 and AMPKα1 mRNAs was decreased in FSP27 KO mice compared with WT mice (Figure 1c). These results suggested that the extent of autophagy is increased in WAT, but attenuated in BAT of FSP27 KO mice. With regard to lipases, the amount of mRNA for adipose triacylglycerol lipase (ATGL), a promoter of autophagy^24^, was increased in eWAT and sWAT of FSP27 KO mice, whereas that of lysosomal acid lipase mRNA was increased in eWAT, and that of hormone‐sensitive lipase mRNA was unaffected in adipose tissues of FSP27 KO mice (Figure 1a,b).

We next examined the expression of autophagy‐related proteins by immunoblot analysis. The amounts of Beclin1 (Atg6), FOXO1, the lipidated form of LC3 (LC3‐II), and both total and phosphorylated (p) forms of AMPKα were increased in eWAT and sWAT, but decreased in BAT of FSP27 KO mice compared with WT mice (Figure 1d). Conversely, the abundance of p62 was decreased in eWAT and sWAT, but increased in BAT of FSP27 KO mice (Figure 1d). Overall, these results were thus consistent with the notion that autophagy is enhanced in WAT and downregulated in BAT of FSP27 KO mice.

### Autophagy is enhanced in a cultured brown adipocyte cell line

The present results also suggested that autophagy is increased in BAT compared with WAT in WT mice (Figure 1d). To test this possibility further, we examined autophagy in HW and HB2 cell lines that were established from p53 KO mice. With regard to expression of FSP27, HW and HB2 adipocytes predominantly expressed FSP27α and FSP27β, respectively (Figure 1e), which are the major isoforms in WAT and BAT, respectively^7^ – indicating that these cell lines retain the characteristics of the parental adipocytes. Immunoblot analysis of serum‐deprived cells with antibodies to LC3 showed that autophagy was increased in HB2 cells compared with HW cells (Figure 1e), consistent with the conclusion that autophagy is enhanced in brown adipocytes.

### Pharmacological inhibition of autophagy attenuates fasting‐induced changes in eWAT weight as well as LD area in white adipocytes of FSP27 KO mice

**Figure 2:**
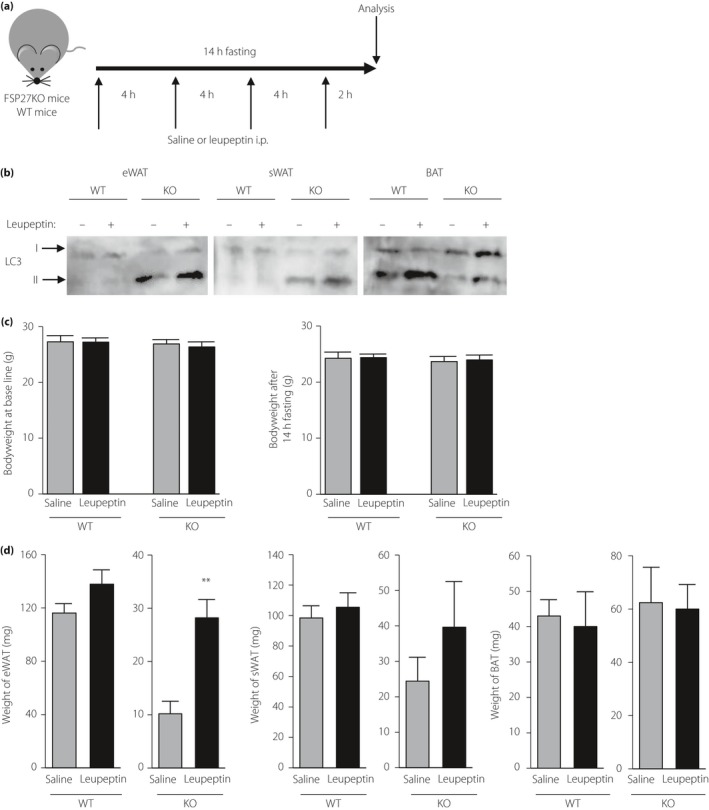
Effects of pharmacological inhibition of autophagy on fasting‐induced changes in body and adipose tissue weight in fat‐specific protein 27 (FSP27) knockout (KO) and wild‐type (WT) mice. (a) Experimental schedule for intraperitoneal (i.p.) injection of leupeptin or saline in 12‐week‐old mice deprived of food. (b) Immunoblot analysis of LC3 in epididymal white adipose tissue (eWAT), subcutaneous WAT (sWAT) and brown adipose tissue (BAT) of WT or FSP27 KO mice at the end of the experimental period. (c) Bodyweight at the baseline and the end of the experimental period and (d) adipose tissue weight of mice at the end of the experimental period. Data are the mean + standard error of the mean (*n* = 5 mice per group). **P <* *0.01 versus the corresponding value for saline.

We previously showed that WAT of FSP27 KO mice is characterized by multilocular LD formation, whereas BAT of these mice conversely manifests large oligolocular LDs^3^.To investigate the effects of autophagy on lipid storage in FSP27 KO mice, we injected food‐deprived WT and mutant mice with saline (control) or with the protease inhibitor, leupeptin, to inhibit autophagy in WAT and BAT (Figure 2a). Consistent with the results shown in Figure 1d, immunoblot analysis with antibodies to LC3 showed that autophagy was enhanced in WAT and attenuated in BAT of saline‐treated FSP27 KO mice compared with WT mice, as well as enhanced in BAT compared with WAT of WT mice (Figure 2b). Injection with leupeptin increased the amount of LC3‐II in each adipose tissue of FSP27 KO mice, as well as in BAT of WT mice (Figure 2b), suggesting that leupeptin inhibited autophagy before breakdown of the contents of autolysosomes. There were no significant changes in bodyweight after 14 h fasting between saline injection and leupeptin injection in WT mice and in FSP27 KO mice (Figure 2c). However, the weight of eWAT at 14 h after the onset of food deprivation was greater in FSP27 KO mice treated with leupeptin than in those injected with saline (Figure 2d). There were no significant differences in the weight of sWAT and BAT between saline injection and leupeptin injection in WT mice and FSP27 KO mice (Figure 2d). In addition, no significant differences were detected in the weight of liver, kidney, heart and gastrocnemius after 14 h fasting between saline injection and leupeptin injection in WT mice and FSP27 KO mice (Figure S1).

**Figure 3:**
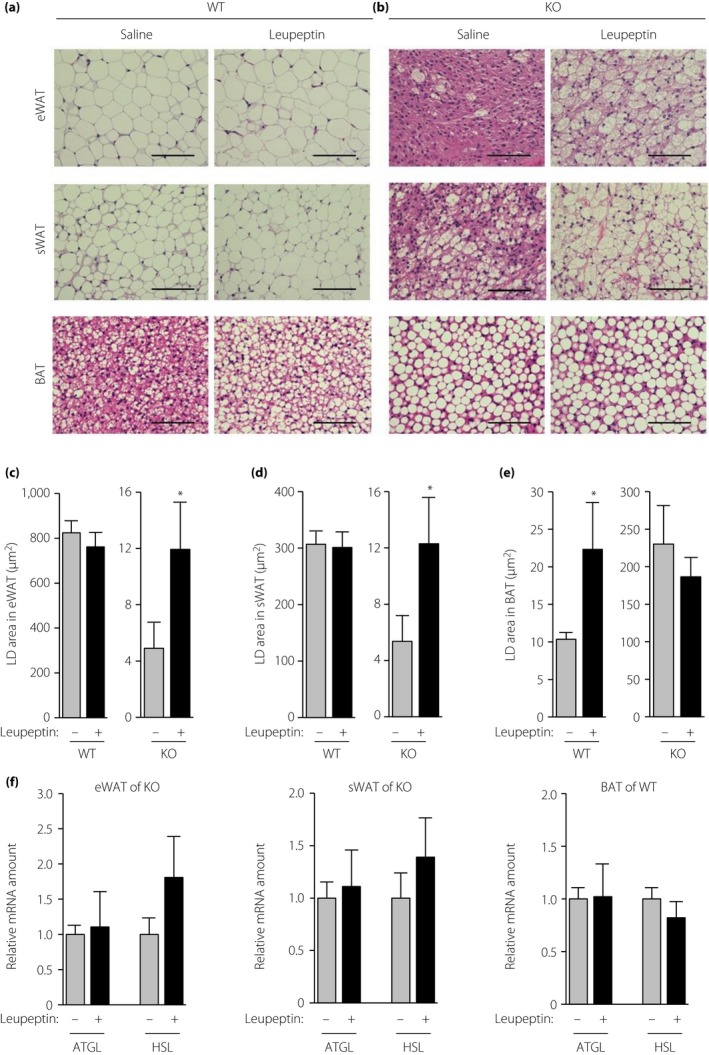
Effects of pharmacological inhibition of autophagy on fasting‐induced changes in lipid droplet (LD) area in white adipose tissue (WAT) and brown adipose tissue (BAT) of fat‐specific protein 27 (FSP27) knockout (KO) and wild‐type (WT) mice. Hematoxylin–eosin staining of sections of epididymal WAT (eWAT), subcutaneous WAT (sWAT) and BAT of (a) WT or (b) FSP27 KO mice treated as in Figure 2a. Scale bars, 100 μm. Mean LD area of (c) eWAT, (d) sWAT and (e) BAT was quantitated from images similar to those in (a) and (b). Data are the mean + standard error of the mean (*n* = 5 mice per group). *P <* *0.05 versus the corresponding value for saline. (f) Quantitative reverse transcription polymerase chain reaction analysis of adipose triacylglycerol lipase (ATGL) and hormone‐sensitive lipase (HSL) messenger ribonucleic acids (mRNAs) in eWAT and sWAT of FSP27 KO mice or in BAT of WT mice as in (a) and (b). Data were normalized by the amount of 36B4 mRNA, are expressed relative to the corresponding value for saline‐treated control mice and are the mean + standard error of the mean (*n* = 5 mice per group).

We next investigated LDs in the treated mice. LD area was greater in eWAT and sWAT of FSP27 KO mice treated with leupeptin than in those injected with saline (Figure 3b,c,d), whereas inhibition of autophagy by leupeptin did not affect LD morphology in WAT of WT mice (Figure 3a,c,d). Conversely, the LD area was greater in BAT of WT mice treated with leupeptin than in those injected with saline (Figure 3a,e). The present results showing that autophagy is augmented in WAT of FSP27 KO mice and in BAT of WT mice thus suggested that increased autophagy might contribute to the fasting‐induced decrease in LD area in cells with multilocular LDs. To exclude the possibility that the effects of leupeptin on LD area in WAT of FSP27 KO mice and in BAT of WT mice were attributable to changes in the expression of cytosolic lipases, we measured the abundance of ATGL and hormone‐sensitive lipase mRNAs. Quantitative RT–PCR analysis showed no significant effects of leupeptin on the amounts of these mRNAs in WAT or BAT of FSP27 KO and WT mice, respectively (Figure 3f).

### Overexpression of FSP27α inhibits autophagy induction by serum deprivation in COS cells

**Figure 4:**
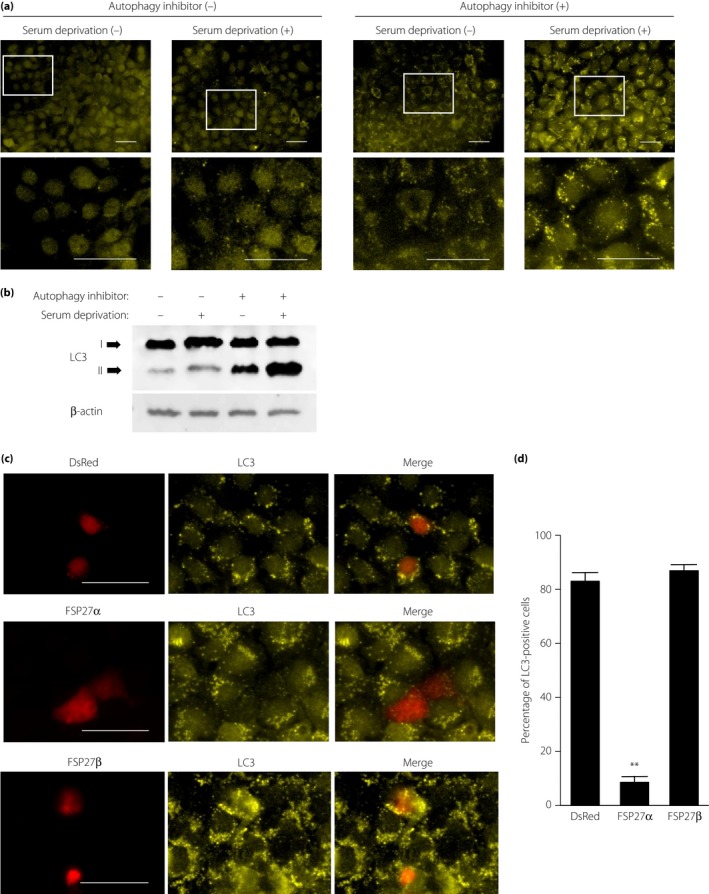
Overexpression of fat‐specific protein 27 (FSP27)α, but not FSP27β, inhibits autophagy induced by serum deprivation in COS cells. (a) COS cells incubated in Dulbecco's modified Eagle's medium with or without 10% fetal bovine serum and 50 μmol/L chloroquine for 18 h were subjected to immunofluorescence staining with antibodies to LC3 and observed with a confocal laser‐scanning microscope. The boxed regions of the upper panels are shown at higher magnification in the lower panels. Scale bars, 50 μm. (b) Immunoblot analysis of LC3 in cells treated as in (a). (c) COS cells were transfected with pIRES2‐DsRed2 encoding (or not, top panels) FSP27α or FSP27β for 2 days, incubated with serum‐free Dulbecco's modified Eagle's medium containing 50 μmol/L chloroquine for 18 h and then subjected to immunofluorescence staining with antibodies to LC3. DsRed2 fluorescence was also observed directly with a confocal laser‐scanning microscope. Scale bars, 50 μm. (d) Quantitation of LC3 staining was carried out by calculating the ratios of LC3‐positive cells in DSRed‐positive cells. Data are the mean + standard error of the mean (*n* = 5). **P <* *0.01 versus the corresponding value for DSRed and FSP27β.

The present results showed that autophagy is restrained in cells with large unilocular LDs, suggesting the possibility that FSP27α, the white adipocyte‐specific isoform of FSP27, might inhibit autophagy. We therefore investigated whether FSP27α can indeed inhibit autophagy in COS cells. We first confirmed that serum deprivation for 18 h markedly increased LC3 immunostaining in the presence of the autophagy inhibitor, chloroquine (Figure 4a), suggesting that serum deprivation induces autophagy in COS cells. Immunoblot analysis with antibodies to LC3 also showed that serum deprivation increased autophagic flow in COS cells (Figure 4b). We then transfected COS cells with an expression vector for both FSP27α and the fluorescent protein, DsRed2, and examined the possible effect of FSP27α overexpression on autophagy induced by serum deprivation. Immunofluorescence analysis showed that LC3 staining was attenuated in serum‐deprived cells overexpressing FSP27α compared with that apparent in cells negative for DsRed2 fluorescence (Figure 4c,d), suggesting that FSP27α inhibited autophagy. In contrast, overexpression of FSP27β had no apparent effect on autophagy (Figure 4c,d). These results might thus account for our observation that autophagy is enhanced in BAT, in which FSP27β is predominantly expressed, compared with that in WAT, in which FSP27α is the major FSP27 isoform.

## Discussion

We have shown that autophagy is enhanced in WAT, especially in eWAT, of FSP27 KO mice and in BAT of WT mice. In addition, overexpression of FSP27α, the major isoform of FSP27 in WAT, inhibited the induction of autophagy by serum derivation in COS cells, whereas overexpression of FSP27β, the major isoform in BAT, had no such effect. Given that WAT in FSP27 KO mice and BAT in WT mice both show multilocular LD formation and contain abundant mitochondria, the enhanced autophagy apparent in these adipocytes might be related to increased metabolic capacity.

Autophagy is an evolutionarily conserved process that is essential for cell survival during periods of nutrient deprivation. Dysregulation of autophagy is associated with various pathologies, including cancer, as well as cardiovascular, neurodegenerative, muscular and metabolic diseases^25^. In addition, the role of autophagy in lipid metabolism is important because of its potential implications for obesity and metabolic syndrome^26^. LDs can be selectively sequestered in autophagosomes and delivered to lysosomes for degradation by lysosomal acid lipases – a process known as lipophagy, which is distinct from classical lipolysis whereby lipids in LDs are directly hydrolyzed by cytosolic lipases without sequestration by autophagosomes^11^. Indeed, targeted inhibition of autophagy in the liver increased hepatic lipid accumulation in mice after starvation or feeding with a high‐fat diet^11^. Furthermore, inhibition of autophagy in hepatocytes also increased cellular TG content during exposure to oleate. The increased lipid storage was characterized by an increase in both the size and number of LDs in the autophagy‐deficient hepatocytes, and it was due to reduced lysosomal lipolysis resulting from decreased delivery of lipid substrates to lysosomes rather than to increased de novo lipogenesis^11^. Lipophagy might therefore play a key role in lipid homeostasis and protect cells against hepatolipotoxicity.

Adipocytes in visceral fat of Atg4b KO mice, which manifest limited autophagic activity, were recently found to undergo a larger increase in size in response to feeding with a high‐calorie diet compared with those in control mice^27^. In addition, pharmacological stimulation of autophagy with spermidine resulted in an increase in the level of autophagy and a decrease in cell size in visceral fat of WT mice^27^. These data suggest that fat content is tightly associated with autophagic activity in fat cells. In contrast, several studies have shown the importance of autophagy in adipocyte differentiation and the beige‐to‐white fat transition^13^, ^14^, ^15^. For example, inhibition of autophagy in 3T3‐L1 preadipocytes, either pharmacologically or by knockdown of Atg5 or Atg7, blocked their differentiation into mature adipocytes and, hence, the increase in their lipid‐storing capacity^13^, ^14^. An adipogenic role for autophagy was further suggested by analysis of adipocyte‐specific Atg7 KO mice, which manifest reduced WAT mass and a lean, insulin‐sensitive phenotype^14^. Loss of WAT mass in these mice was accompanied by increased BAT‐like features in white adipose depots^13^. Given that these various studies were carried out with undifferentiated adipocytes or with mice generated by germline genetic manipulation, it was difficult to evaluate the effects of autophagy alone on the physiology of mature adipocytes independent of the effect on adipocyte differentiation. However, a recent study showed that cold exposure activated autophagy in BAT of control mice, and that prevention of autophagy by genetic ablation of Atg7 specifically in BAT of adult mice reduced LD turnover and blocked lipophagy^28^. Conversely, activation of autophagy induced breakdown of LDs in BAT^28^. In the present study, we inhibited autophagy pharmacologically in adult mice in order to exclude the effect of such inhibition on BAT differentiation. Our finding that inhibition of autophagy attenuated the fasting‐induced disappearance of LDs in BAT is consistent with the previous observation that inhibition of autophagy in BAT reduced the cold‐induced degradation of LDs^28^.

Although the precise mechanism by which FSP27α inhibits autophagy remains to be elucidated, several mechanisms are assumed. AMPK is a major activator of autophagy that is mediated through the mammalian target of rapamycin and Ulk1^23^, ^29^. FSP27α decreases the expression of AMPK through the ubiquitin/proteasome pathway in cultured cells^30^. This reduction of AMPK is one of the possible mechanisms that might underlie the inhibitory effect of FSP27α on autophagy. In addition, ATGL promotes autophagy through SIRT1^24^. Given that FSP27α interacts with ATGL and inhibits its activity through perilipin in human adipocytes^31^, FSP27α might inhibit autophagy through the interaction of ATGL. FSP27β is a molecule to which 10 amino acids were added to the amino‐terminal domain of FSP27α^8^. However, these two molecules show the opposite effects to the LD formation. FSP27α forms a homodimer and induces unilocular large LD formation in white adipocytes^6^, ^32^, ^33^, whereas FSP27β promotes multilocular small LD formation by inhibiting the homodimerization of CideA in brown adipocytes^7^. Therefore, 10 amino acids in the amino‐terminal domain of FSP27β are supposed to play a critical role in LD formation through the interaction with other molecules. Given the possibility that FSP27α might inhibit autophagy by interacting with other molecules, as mentioned above, FSP27β might not be able to show the inhibitory effects because of the alteration of the steric structure induced by the 10 amino acids in its amino‐terminal domain.

The enhancement of fasting‐induced autophagy in WAT of FSP27 KO mice and BAT of WT mice suggests that autophagy might be associated with the presence of multilocular LDs and abundant mitochondria, both of which are characteristics of metabolically active, energy‐consuming adipocytes. FFAs generated in energy‐consuming BAT are taken up by mitochondria for beta‐oxidation. In contrast, FFAs generated in energy‐storing WAT are delivered to other tissues through the circulation for utilization as an energy substrate. Lipolysis through autophagy might promote the effective flux of FFAs into mitochondria in cells with multilocular LDs and thereby contribute to energy expenditure in metabolically active cells. Indeed, BAT from cold‐exposed mice in which autophagy was inhibited by Atg7 ablation manifested an ~50% reduction in the rate of oxygen consumption^28^. In addition, oxygen consumption was previously shown to be reduced in BAT of FSP27 KO mice^3^, in which we have now shown that autophagy is attenuated. FSP27α might thus contribute to the metabolic characteristics of energy‐storing white adipocytes by inhibiting lipophagy, as well as by promoting large unilocular LD formation. These findings might elucidate the underlying mechanism of the different energy metabolism between white adipocytes and brown adipocytes.

## Disclosure

The authors declare no conflict of interest.

## Supporting information


**Figure S1** ¦ Effects of pharmacological inhibition of autophagy on fasting‐induced changes in liver, kidney, heart, and gastrocnemius weight in WT and FSP27 KO mice.
