# Supplementary figures and images for: Fat‐specific protein 27α inhibits autophagy‐dependent lipid droplet breakdown in white adipocytes
Source: J Diabetes Investig. 2019 Apr 26;10(6):1419–29. doi: 10.1111/jdi.13050 (PMC6825946; doi:10.1111/jdi.13050)

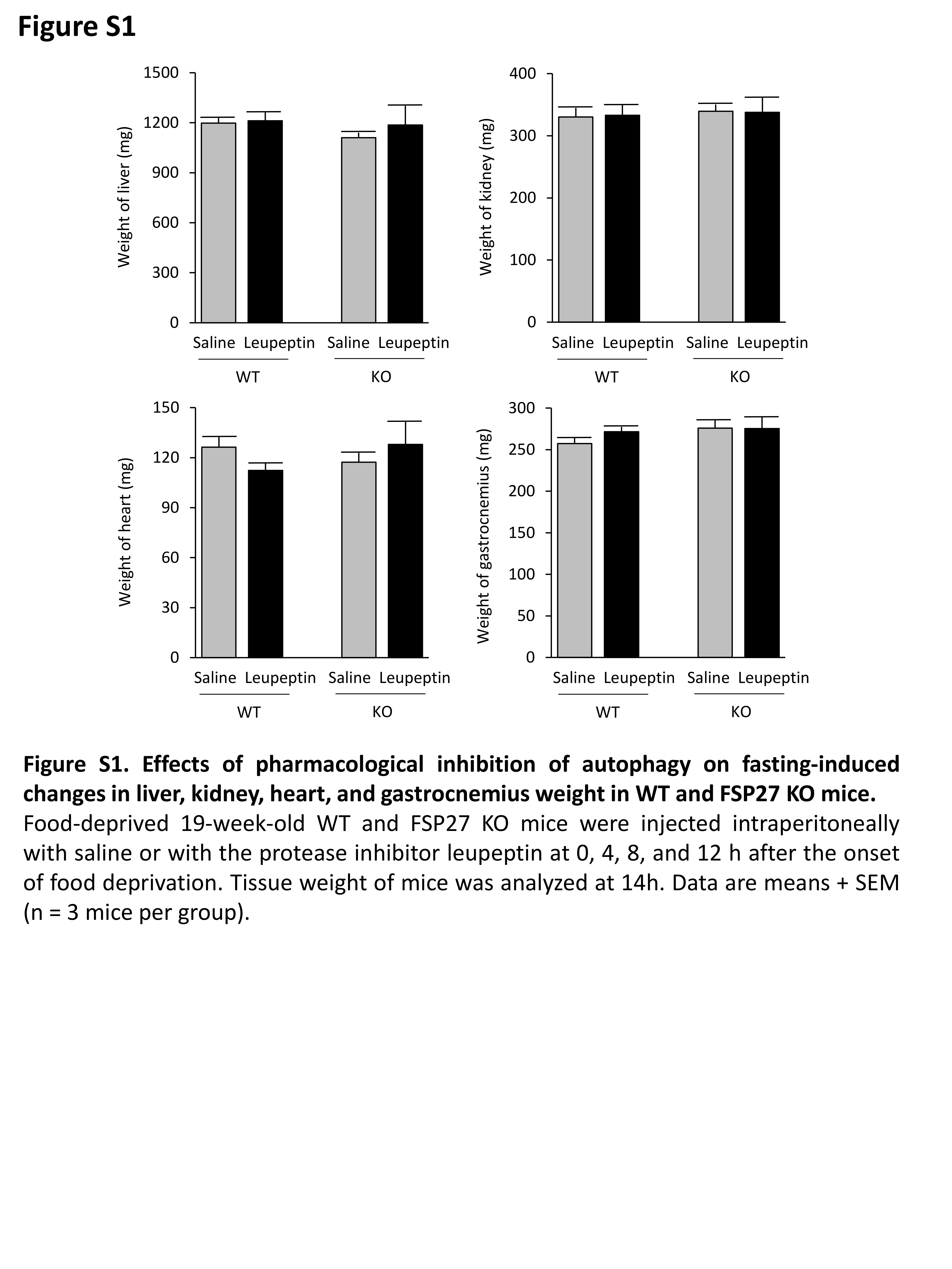

Supplement: Supplementary file 1 — Figure S1 ¦ Effects of pharmacological inhibition of autophagy on fasting‐induced changes in liver, kidney, heart, and gastrocnemius weight in WT and FSP27 KO mice. [file JDI-10-1419-s001.jpg]
